# Fabrication and Characterization of Konjac Glucomannan/Oat β-Glucan Composite Hydrogel: Microstructure, Physicochemical Properties and Gelation Mechanism Studies

**DOI:** 10.3390/molecules27238494

**Published:** 2022-12-02

**Authors:** Xiaoyuan Geng, Nuo Zhao, Xiwang Song, Jianfu Wu, Qiaomei Zhu, Tao Wu, Haixia Chen, Min Zhang

**Affiliations:** 1 State Key Laboratory of Food Nutrition and Safety, Tianjin University of Science & Technology, Tianjin 300457, China; 2 Tianjin Key Laboratory for Modern Drug Delivery & High-Efficiency, School of Pharmaceutical Science and Technology, Tianjin University, Tianjin 300072, China; 3 China-Russia Agricultural Processing Joint Laboratory, Tianjin Agricultural University, Tianjin 300392, China

**Keywords:** polysaccharide, composite hydrogel, gel properties, gelation mechanism

## Abstract

The aim of this study was to evaluate the effect of oat β-glucan on the formation mechanism, microstructure and physicochemical properties of konjac glucomannan (KGM) composite hydrogel. The dynamic rheology results suggested that the addition of oat β-glucan increased the viscoelastic modulus of the composite hydrogel, which was conducive to the formation of a stronger gel network. Gelling force experiments showed that hydrogen bonds and hydrophobic interactions participated in the formation of the gel network. Textural profile analysis results found that the amount of oat β-glucan was positively correlated with the elasticity, cohesiveness and chewiness of the composite hydrogel. The water-holding capacity of the composite hydrogel was enhanced significantly after the addition of oat β-glucan (*p* < 0.05), which was 18.3 times that of the KGM gel. The thermal stability of KGM gel was enhanced after the addition of oat β-glucan with the increase in Tmax being approximately 30 °C. Consequently, a composite hydrogel based on KGM and oat β-glucan was a strategy to overcome pure KGM gel shortcomings.

## 1. Introduction

Hydrogel, absorbing a large quantity of water into porous polymer networks by physical or chemical crosslinking, is a kind of three-dimensional network [1] which is extensively applied in food technology, biomedicine, cosmetics, agriculture and so on [2]. Hydrogel can be divided into single hydrogel prepared by one ingredient and composite hydrogel prepared by two ingredients or more. The mono-hydrogel provides the basic information of the product in terms of physicochemical properties; however, the mechanical properties of the mono-hydrogel are relatively weak [3]. Compared with single hydrogel, composite hydrogel can enhance gel properties. Typically, composite hydrogel has a flexible structure, which can be adjusted by polymer type, concentration, proportion and processing manners [4]. Konjac glucomannan, oat β-glucan, carrageenan and sodium alginate are common gel materials and are often used in conjunction with other hydrocolloids to exert synergistic effects in the field of food.

Konjac glucomannan (KGM) is separated from the roots of Amorpho phallus konjac [5], which is composed of mannose and glucose by β-1,4 linkages and a small amount of acetyl groups at the C-6 position [6]. KGM owns significant health benefits such as reducing cholesterol, regulating blood sugar levels, antiobesity, anti-inflammatory and promoting the absorption of vitamins [7]. It is reported that KGM also holds excellent functional properties including thickening, texturing, gelling and water-binding capability as a kind of food additive [8]. In addition, it is worth noting that KGM can form gel at low concentrations [9]. Generally, there are two approaches to fabricating KGM gel: (a) heating KGM solution to form a thermally reversible gel, which is applied in low temperature foods and (b) thermal alkali treatment forms a thermally irreversible gel, which can resist high temperature and is suitable for high temperature foods [10]. However, KGM gel has the disadvantages of higher cooking loss, poor water-holding capacity and low emulsion stability, which limits further application in the food industry [11].

Oat β-glucan is a nonstarch polysaccharide existing in cell walls of grain, which is widely used as animal feed or waste. Oat β-glucan is composed of glucose linked β-(1-3) and β-(1-4) glycosidic bonds [12]. Lots of studies have shown that oat β-glucan possesses multiple biological benefits. For example, oat β-glucan is able to reduce postprandial blood glucose, decrease insulin response and regulate bile acid absorption [13]. Oat β-glucan is also an excellent antioxidant, which can reduce the damage of oxidative stress to the human body by scavenging free radicals [14]. In addition, oat β-glucan has a strong viscosity, bland (neutral) flavor, and can form molecular aggregates and network structures, so it is widely used in the food industry as a kind of hydrophilic colloid [15].

In recent years, composite hydrogel composed of KGM and xanthan, flaxseed gum or k-carrageenan have played an important role in the food industry to overcome the limitations of KGM gel. Oat β-glucan is one of the important polysaccharides in the food industry. Therefore, the composite hydrogel of KGM/oat β-glucan might not only exert the advantages of synergistic gel, but also effectively solve the problems of obvious water separation and poor texture performance of the pure KGM gel.

In this study, we firstly studied the influence of KGM mixed with oat β-glucan on the hydrocolloidal properties. The aim of this study was to evaluate the effects of oat β-glucan on the formation mechanism, microstructure and physicochemical properties of konjac glucomannan (KGM) composite hydrogel. The rheological properties, textural profile analysis, microstructures and thermodynamic properties of composite hydrogel were measured to better understand the gelation mechanism, aiming to design KGM gel with great water-holding capacity, outstanding mechanical properties and excellent thermal stability.

## 2. Results

### 2.1. Gel Evolution Process

#### 2.1.1. Temperature Sweep

**Figure 1:**
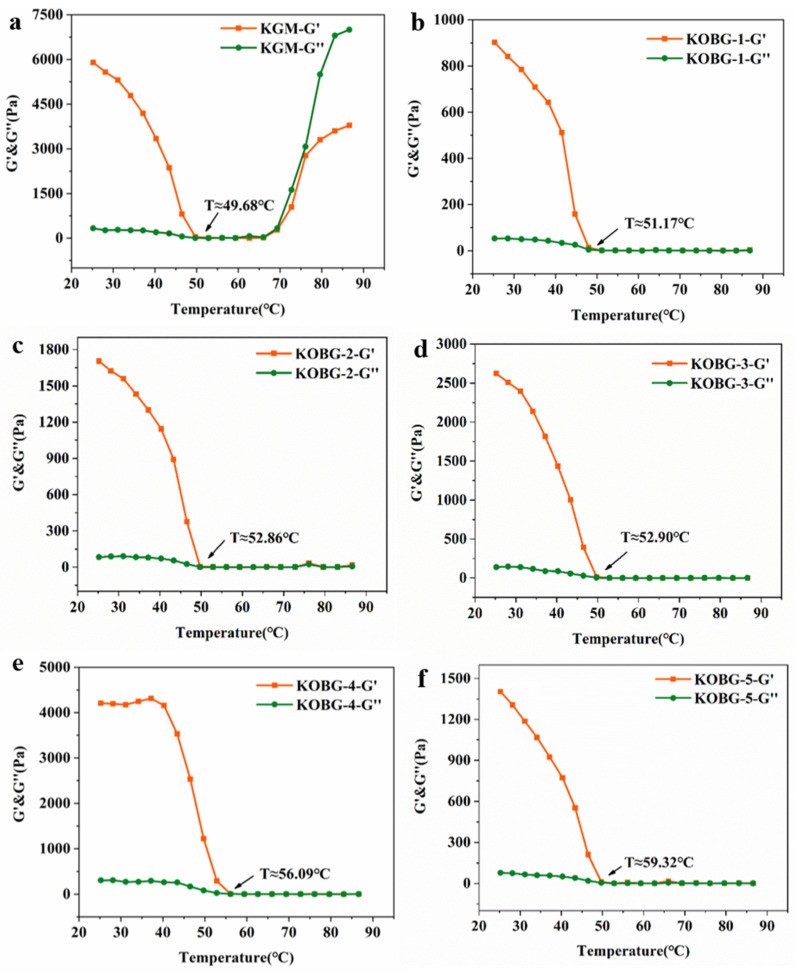
Temperature sweep results of konjac glucomannan (KGM) gel (**a**), KOBG-1 (**b**), KOBG-2 (**c**), KOBG-3 (**d**), KOBG-4 (**e**) and KOBG-5 (**f**). KOBG-1: 1% oat β-glucan; KOBG-2: 2% oat β-glucan; KOBG-3: 3% oat β-glucan; KOBG-4: 4% oat β-glucan; KOBG-5: 5% oat β-glucan.

Temperature sweep was detected to monitor the gel evolution process under programmed cooling. As shown in Figure 1, the loss modulus (G″) and storage modulus (G′) of KGM gel emerged descending trends and the values of G′ and G″ were closer when cooling to around 50 °C, which indicated that the KGM solution was in sol state at this point. The gelation temperature of KGM gel was 49.68 °C. As the temperature decreased from 49.68 °C to 25 °C, the G′ showed a noticeable increase and the G″ kept a straight line, showing the formation of a strong gel network [16]. For the composite hydrogel, the G′ and G″ kept steady during the approach from 90 °C to 50 °C, which was different with the trend of pure KGM gel. This phenomenon might be due to the enhanced intermolecular interaction between KGM and oat β-glucan [17]. In addition, the gelation temperature of composite hydrogel with 1% oat β-glucan (KOBG-1), composite hydrogel with 2% oat β-glucan (KOBG-2), composite hydrogel with 3% oat β-glucan (KOBG-3), composite hydrogel with 4% oat β-glucan (KOBG-4) and composite hydrogel with 5% oat β-glucan (KOBG-5) were 51.17 °C, 52.86 °C, 52.90 °C, 56.09 °C and 59.32 °C, respectively. The gelation temperature of the composite hydrogel increased with the amount of oat β-glucan, indicating that the addition of oat β-glucan improved the KGM gel process.

#### 2.1.2. Frequency Sweep

**Figure 2:**
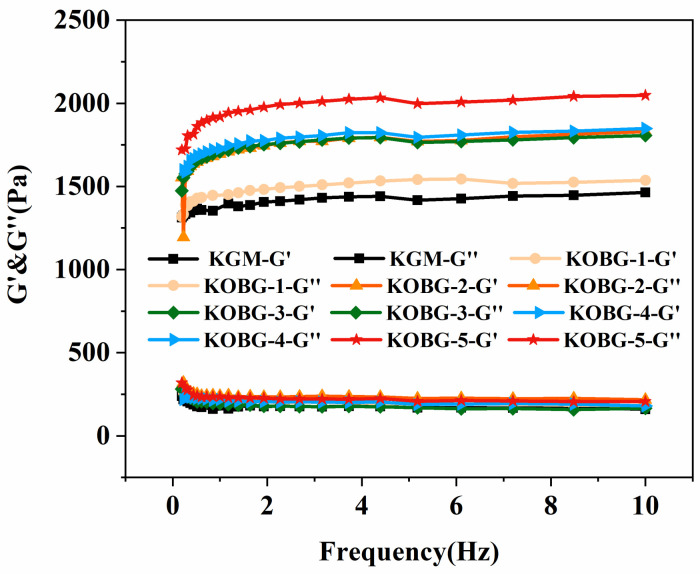
Frequency sweep results of KGM gel and composite hydrogel.

Frequency sweep was conducted to observe the viscoelastic property of KGM gel and composite hydrogel. As shown in Figure 2, the G′ values were higher than the G″ values in all sample gels, indicating the formation of a strong gel network. The G′ values of the KGM gel and the composite hydrogel kept a smooth straight line, and they were parallel to each other. The values did not change with frequency, which might be due to the high elasticity. Addition of oat β-glucan increased the G′ value of the composite hydrogel and was positively correlated with the concentration, which was consistent with previous research results [18]. Oat β-glucan is rich in hydroxyl groups, which enhances the binding with other hydrophilic polysaccharides. In this study, the addition of oat β-glucan increased the G′ values and formed a more resilient gel network.

### 2.2. Gelling Force

#### 2.2.1. Interaction Force Test

**Figure 3:**
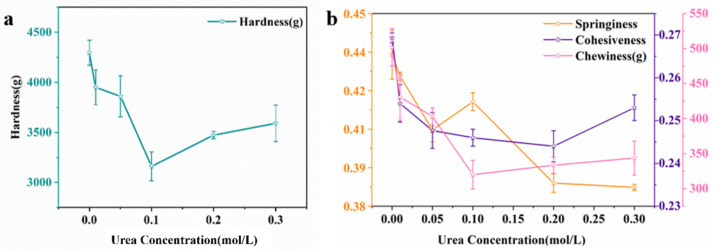
Textural profile analysis (TPA) parameters of hardness of composite hydrogel with different urea concentrations (**a**); springiness, cohesiveness and chewiness of composite hydrogel with different urea concentrations (**b**).

As a kind of representative composite hydrogel, KOBG-4 was selected for the interaction force test. Various driving forces during gel formation were analyzed by adding different denaturants, including hydrogen bonds and hydrophobic interactions. According to the previous reports, urea can destroy hydrogen bonds and sodium dodecyl sulfate (SDS) can destroy hydrophobic interactions [19,20]. As shown in Figure 3a,b, the hardness of KOBG-4 showed a downward trend and had the lowest value at 0.1 mol/L urea. Subsequently, the hardness of KOBG-4 showed an upward trend with the addition of urea, which was due to the molecular rearrangement of oat β-glucan and KGM. This enabled the texture characteristics’ recovery. The traces of springiness, cohesiveness and chewiness were consistent with the hardness curve. Therefore, urea could disrupt the gel network, and hydrogen bonds played an important role in the composite hydrogel system.

**Figure 4:**
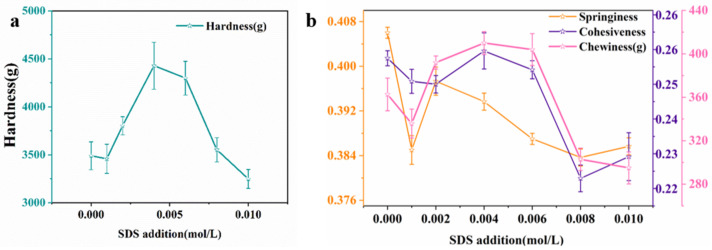
TPA parameters of hardness of composite hydrogel with different sodium dodecyl sulfate (SDS) concentrations (**a**); springiness, cohesiveness and chewiness of composite gel with different SDS concentrations (**b**).

The hardness of KOBG-4 first increased and then decreased with the addition of SDS in Figure 4. The springiness, cohesiveness and chewiness had similar tendency. This might be due to the interaction between SDS and KGM, resulting in the strengthening of the texture characteristics of composite hydrogel. Some research implied that the main forces of KGM gel were hydrogen bonds and hydrophobic interactions, and the ability of hydrogen bonds were decreased with temperature [10]. SDS could bind the hydrophobic microregion of the KGM gel. So, hydrophobic interactions played an important role in the composite hydrogel.

#### 2.2.2. Fourier Transform Infrared Spectroscopy Analysis (FT-IR)

**Figure 5:**
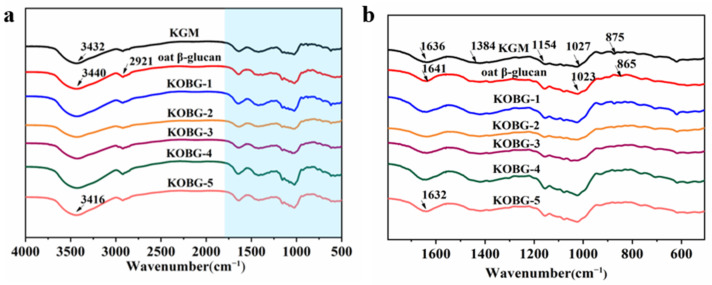
Fourier transform infrared spectroscopy (FT-IR) of composite hydrogel at 4000–500 cm^−1^ (**a**); enlarged FT-IR of composite hydrogel at 1800–600 cm^−1^ (**b**).

FT-IR is an important means to detect whether composite hydrogel is successfully fabricated. Figure 5a,b shows FT-IR spectra of KGM, oat β-glucan and the composite hydrogel. For the FT-IR spectra of oat β-glucan, the broad peak at 3440 cm^−1^ corresponded to the stretching vibration of O–H and the peak at 2921 cm^−1^ was attributed to the stretching vibration of C–H in the sugar ring. The peak at 1641 cm^−1^ was attributed to the stretching vibration of C=O and –CHO. The peak at 1023 cm^−1^ represented the stretching vibration of C–O–C. The band at 865 cm^−1^ was the stretching vibration of C–H in β-D-pyranose [21]. For the KGM gel, the absorption bands at 3432 cm^−1^ and 1636 cm^−1^ were attributed to the stretching and bending vibrations of –OH, respectively [22]. The absorption band at 1384 cm^−1^ was ascribed to the C–H bending vibration of methyl from the acetyl group, and the absorption bands at 1154 cm^−1^ and 1027 cm^−1^ were attributed to the stretching vibration of C–O–C. The absorption band at 875 cm^−1^ was ascribed to the glucose and mannose stretching vibration [23].

The incorporation of oat β-glucan into the KGM gel led to the shifting of the peak at 3432 cm^−1^ to 3416 cm^−1^ because of the strengthened hydrogen bonding interaction. The peak at 1636 cm^−1^ shifted right to 1632 cm^−1^ proved that the composite hydrogel formed stronger hydrogen bonds both intermolecular and intramolecular. The band at 1154 cm^−1^ increased in intensity because of C–O stretching vibration and C–OH bending. From the peak of 1027 cm^−1^ to 1023 cm^−1^, the absorption peak became weaker due to hydrogen bond coupling. In addition, the peak at 875 cm^−1^ was weak, which indicated the stronger intermolecular interaction. The results from FT-IR indicated that KGM and oat β-glucan were mainly crosslinking by hydrogen bonds and the composite hydrogel were fabricated successfully.

### 2.3. Scanning Electron Microscopy Observations (SEM)

**Figure 6:**
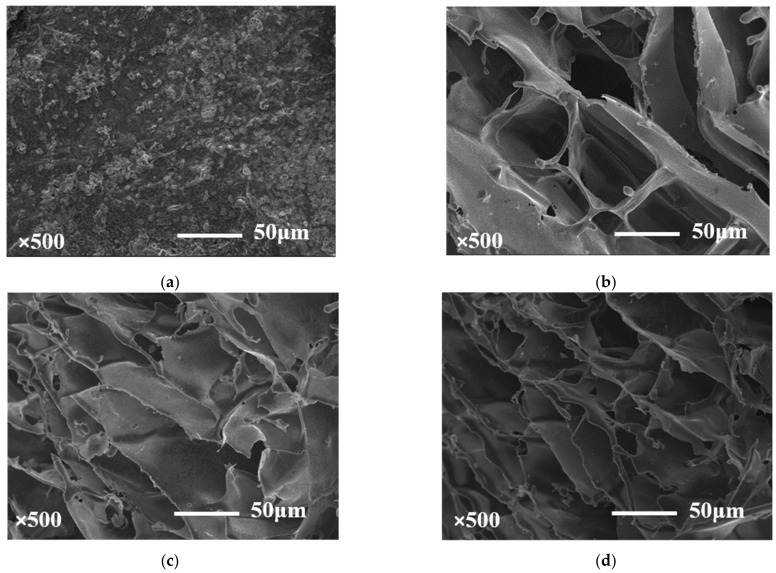

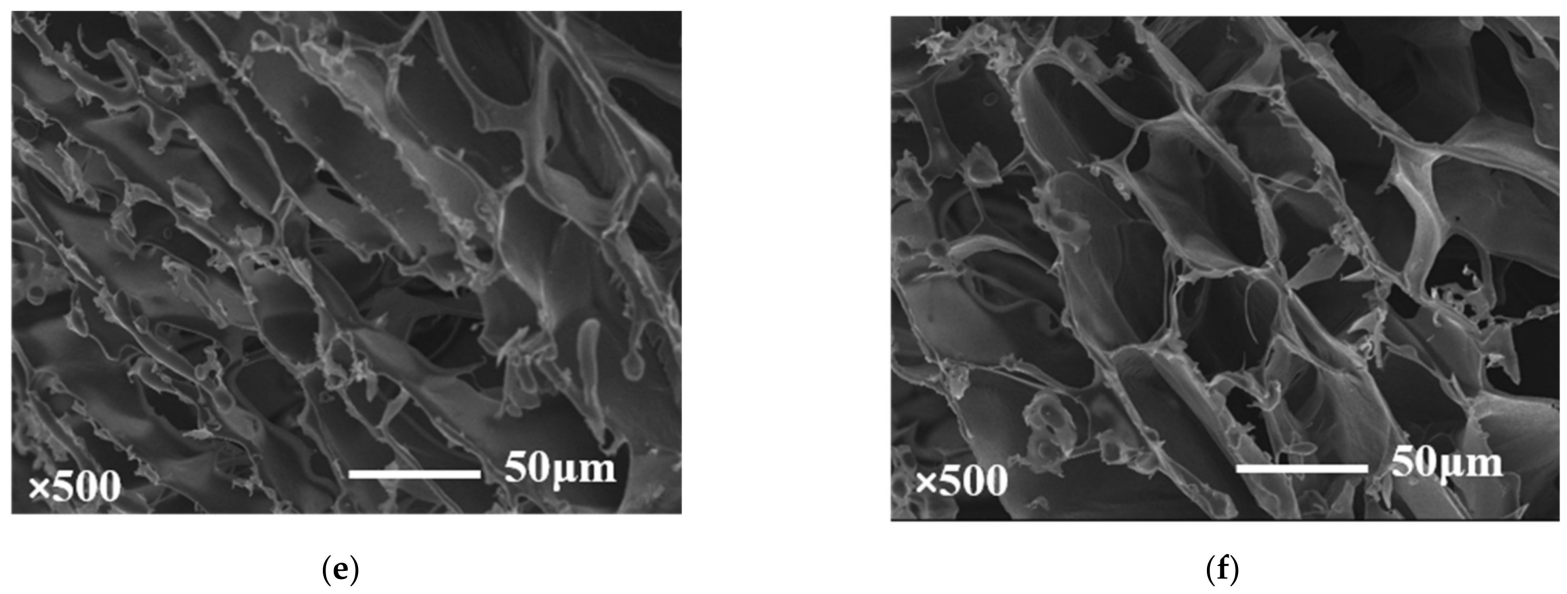
Scanning electron microscopy observations (SEM) of KGM gel (**a**), KOBG-1 (**b**), KOBG-2 (**c**), KOBG-3 (**d**), KOBG-4 (**e**) and KOBG-5 (**f**).

As shown in Figure 6, the microstructures of the composite hydrogel and pure KGM gel were displayed at a magnification of 500×. Pure KGM gel showed an accumulated flake aggregate structure similar to Tao Zhang [24], which was related to the deacetylation of the KGM-formed self-coiling structure under the condition of alkali treatment. In addition, KGM gel was the main gel skeleton of the composite hydrogel, which was ascribed to the fact that the oat β-glucan solution did not form a network structure [25]. Compared with KGM gel, the composite hydrogel exhibited honeycomb network structures. The sample of KOBG-1 showed a sheet-like network structure with large pore size and the samples of KOBG-2, KOBG-3, KOBG-4 and KOBG-5 exhibited smaller pore size network structures with further addition of oat β-glucan. The reason for the above phenomenon might be the formation of a double physical crosslinked network structure, including the fact that KGM molecules themselves formed a network structure by hydrogen bonds and hydrophobic interactions, and KGM and oat β-glucan formed a network structure by hydrogen bonds at the same time. In addition, as the concentration of oat β-glucan increased, the degree of crosslinking between the oat β-glucan molecules and KGM molecules increased, resulting in a denser gel network structure and a smaller pore size [9].

### 2.4. Physicochemical Properties

#### 2.4.1. Textural Profile Analysis (TPA)

**Figure 7:**
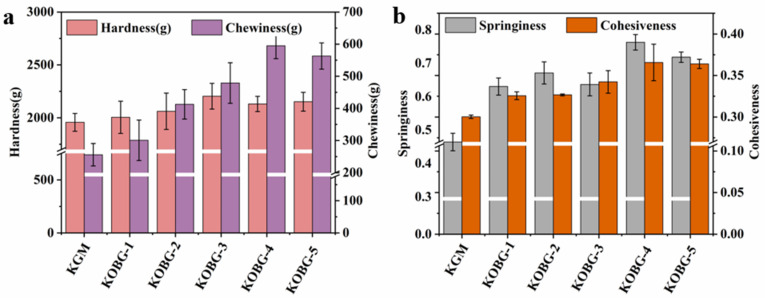
Hardness and chewiness of composite hydrogel on different oat β-glucan additions (**a**); springiness and cohesiveness of composite hydrogel on different oat β-glucan additions (**b**).

Hardness represents the force required by the teeth and palate to compress food. Springiness represents the potential of gel to resist the pressure of the external environment. Cohesiveness refers to the degree of compression of a material before it is destroyed. Chewiness is the energy required to swallow the solid food, which is related to hardness, springiness and cohesiveness [26]. Figure 7a,b shows the texture properties’ profiles of KGM gel and composite hydrogel, and there are significant differences in chewiness, elasticity and cohesion. There was no significant difference in the hardness between KGM gel and the composite hydrogel (Figure 7a). It was obvious that the chewiness, springiness and cohesiveness of the composite hydrogel had significant increases after the rise of the oat β-glucan concentration. The chewiness, springiness and cohesiveness of the KOBG-4 sample were the highest, which were significantly higher than that of the pure KGM gel. Typically, the increase of gel textural properties was associated with the degree of crosslinking, which could be attributed to stronger intermolecular interaction. It was reported that concentration and crosslinking degree of polysaccharide molecules affects the textural properties of gels [9]. Thus, it was deduced that the interaction between oat β-glucan and KGM molecules was enhanced to form a denser gel network. In addition, the addition of a high concentration of oat β-glucan molecules reduced the space area of water molecules and further strengthened the gel network structure. The honeycomb network structure had the following two advantages. First, it was favorable for the delivery of water-soluble nutrients. Second, it was advantageous to trap more moisture. So, the microstructure and functionality of the oat β-glucan and KGM composite hydrogel could aid in food applications.

#### 2.4.2. Water-Holding Capacity (WHC) of Composite Hydrogel

**Figure 8:**
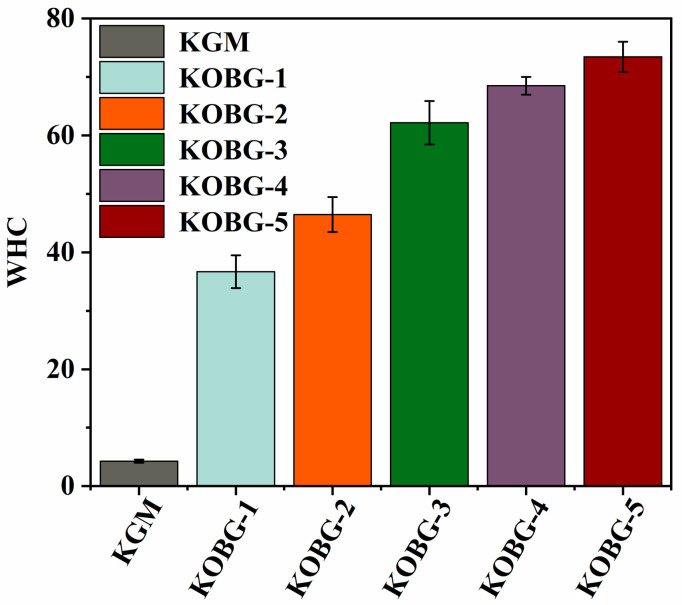
Water-holding capacity (WHC) of composite hydrogel.

WHC is an important parameter to evaluate the level of combination of gel and water. It was clear from the images in Figure 8 that the WHC of the composite hydrogel was significantly higher than that of the KGM gel (*p* < 0.05). When the concentration of oat β-glucan was 5%, the WHC of KOBG-5 was 73.4%, which was 18.3 times that of the KGM gel. It is widely believed that the dense gel network structure traps a large amount of water, leading to an increased flooding rate. On the one hand, the oat β-glucan formed a dense gel network by physical crosslinking with KGM to trap more water molecules. On the other hand, the hydroxyl groups in oat β-glucan molecules formed hydrogen bonds with H in water, which also was beneficial to improving the water-holding capacity of the composite hydrogel. Therefore, the addition of oat β-glucan improved the WHC of KGM gel. In addition, higher WHC is also beneficial for the food industry, because it can resist quality loss of products in extreme environments. Tofu products with high WHC had outstanding morphology, texture, and cooking characteristics. Meat products with high WHC possessed excellent juiciness and tenderness, but low WHC made meat products weak in acceptability.

#### 2.4.3. Thermogravimetric Analysis (TGA)

**Figure 9:**
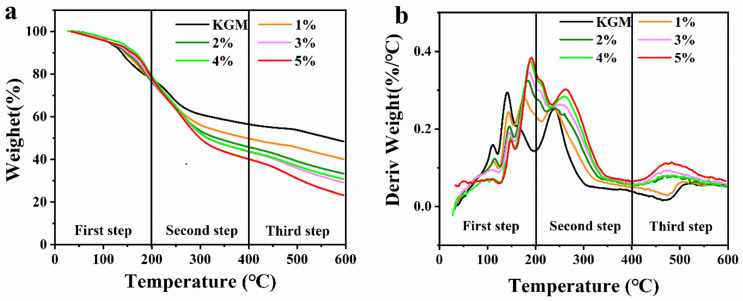
Thermogravimetric analysis (TGA) of samples (**a**); differential thermogravimetric (DTG) of samples (**b**).

Figure 9 and Table 1 show the thermal stability profiles of pure KGM gel and composite hydrogel using TGA curves and differential thermogravimetric (DTG) curves. There were three steps of weight loss of all samples between 30 °C and 600 °C. All samples offered the same weight loss in the first step from 30 °C to 200 °C. In addition, the weight loss of the first step was ascribed to the vaporization of bound water and solvent [27]. The second step of weight loss was related to the thermal degradation of the gel network between 200 °C and 400 °C. The weight loss of KGM gel, KOBG-1, KOBG-2, KOBG-3, KOBG-4 and KOBG-5 was 20.94%, 27.74%, 31.5%, 33.76%, 33.78% and 37.43%, respectively, which was due to the increased addition of polysaccharides. It was noteworthy that the temperatures of the maximum weight loss were observed from the DTG curves in a sequence of 238.75 °C, 239.13 °C, 240.37 °C, 244.73 °C, 259.49 °C and 261.86 °C, respectively. This phenomenon might be due to the fact that the incorporation of oat β-glucan increased the dense network structure of KGM gel, and the hydrogen bond and hydrophobic bonds formed between polysaccharides increased the spatial resistance, resulting in the improvement of the thermal stability of the composite hydrogel. This was consistent with the results of FT-IR. The third step of weight loss of the KGM gel and the composite hydrogel was due to further decomposition of saccharide rings from 400 °C to 600 °C [28]. Thus, it was presumed that the addition of oat β-glucan enhanced the thermal stability of the KGM gel.

## 3. Materials and Methods

### 3.1. Materials

Konjac glucomannan was obtained from Hubei Konson Konjac Technology Co., Ltd. (Wuhan, China). Oat β-glucan (M_W_ = 500 kDa) was purchased from Shaanxi Yuanbeichun Technology Co., Ltd. (Xi’an, China). All the chemical reagents were analytical grade and were purchased from Sinopharm Chemical Reagent Co., Ltd. (Shanghai, China).

### 3.2. Fabrication of KGM/Oat β-Glucan Composite Hydrogel

The composite hydrogels with different series of concentrations were fabricated according to Yang X. [16], followed by pure KGM gel, KGM gel with 1% (*w*/*v*) oat β-glucan, KGM gel with 2% (*w*/*v*) oat β-glucan, KGM gel with 3% (*w*/*v*) oat β-glucan, KGM gel with 4% (*w*/*v*) oat β-glucan, and KGM gel with 5% (*w*/*v*) oat β-glucan, which were defined as KGM, KOBG-1, KOBG-2, KOBG-3, KOBG-4, and KOBG-5, respectively. For pure KGM gel, the KGM powder was dissolved in distilled water to obtain 2% (*w*/*v*) solution, which was stirred magnetically for 4 h at 90 °C. KGM solution was added to 0.5% (*w*/*v*) Na_2_CO_3_ and incubated 10 min to form an irreversible gel. For the composite hydrogel, the oat β-glucan powder was hydrated in distilled water to obtain a series of stock solutions, followed by 1% (*w*/*v*), 2% (*w*/*v*), 3% (*w*/*v*), 4% (*w*/*v*) and 5% (*w*/*v*), which were stirred for 30 min at 100 °C. Then, the KGM stock solution and oat β-glucan stock solution were mixed together and added to 0.5% (*w*/*v*) Na_2_CO_3_ to incubate for 10 min. Finally, we cooled all the samples to room temperature for further experiments.

### 3.3. Monitoring of Gel Evolution Process

#### 3.3.1. Linear Viscoelastic Region Measurement (LVR)

The LVR was determined by the amplitude sweep. The strain of all the samples varied from 0.1–10% with frequency of 1 Hz. The amplitude sweep presented that a deformation of 0.5% was within LVR.

#### 3.3.2. Temperature Sweep

Gel evolution process was measured by Rheometer (MARS 60, German thermal power company, Germany) with a method according to Yang et al. [16]. The parallel plate geometry was of the diameter 20 mm and gap 1 mm. The KGM solution and oat β-glucan were mixed and added to 0.5% (*w*/*v*) Na_2_CO_3_. The mixed solution was incubated in a 90 °C water bath. A total of 0.4 mL of solution was added to the rheometer and the parallel plate was adjusted to 1 mm. The edge of the parallel plate was sealed with silicone oil to prevent water evaporation. The temperature sweep measurements were conducted from 90 °C to 25 °C at a cooling rate of 2 °C/min and 1 Hz. The storage modulus G′ and loss modulus G″ were recorded.

#### 3.3.3. Frequency Sweep

All samples were cut into 1 mm and placed on the parallel plate geometry. The frequency sweep measurements were determined ranging from 0.1 Hz to 10 Hz at a shear strain of 0.5%. The storage modulus G′ and loss modulus G″ were recorded.

### 3.4. Gelling Force

#### 3.4.1. Interaction Force Test

Variations of the TPA parameters can evaluate the interaction force between KGM and oat β-glucan as described by Qian Ju [29]. Urea (0.000 mol/L, 0.05 mol/L, 0.1 mol/L, 0.2 mol/L, 0.3 mol/L) and SDS (0.000 mol/L, 0.001 mol/L, 0.002 mol/L, 0.004 mol/L, 0.006 mol/L, 0.008 mol/L, 0.01 mol/L) were added into the composite hydrogel. The composite hydrogel was fabricated at a fixed concentration of KGM (2% *w*/*v*) and at a fixed concentration of oat β-glucan (4% *w*/*v*). The chemical bonds were reflected by the varieties of TPA parameters.

#### 3.4.2. Fourier Transform Infrared Spectroscopy Analysis (FT-IR)

The FT-IR spectrum of the composite hydrogel was determined on a Nicolet Is50 FT-IR spectroscope (Thermo Fisher Scientific, Madison, WI, USA). The sample film was recorded from the wavenumbers of 4000 cm^−1^ to 400 cm^−1^ and was scanned 16 times.

### 3.5. Scanning Electron Microscopy Observations (SEM)

The morphology of the composite hydrogel was elucidated using a scanning electron microscope (Hitach, Japan) at an accelerating voltage of 10 KV. Before the test, the samples were sliced and freeze-dried. All the samples were stuck on the board with double sticky tape and sputter-coated with gold. The microstructure of the composite hydrogel was observed at a magnification of 500×.

### 3.6. Physicochemical Properties

#### 3.6.1. Textural Profile Analysis (TPA)

The texture parameters of the composite hydrogel were analyzed by TA-XT plus/30 (Stable Micro Systems, Surrey, UK) using a cylinder probe (P100). The test sample was cut into cubes with a length of 2 cm, a width of 2 cm, and a height of 2 cm. The test pattern was TPA. The speed was 4 mm/s before the test and 2 mm/s during the test. The speed was 2 mm/s after the test. The compression deformation was 50%. Texture characters including hardness, springiness, chewiness and cohesiveness were recorded.

#### 3.6.2. Water-Holding Capacity (WHC)

The WHC parameters of the composite hydrogel were determined by the centrifugal method [30]. The initial weights of the samples were recorded, and the composite hydrogels were centrifuged at 3000× *g*, 4 °C for 15 min. Then, the water was dried, and the final weights of the samples were recorded. The WHC (%) was defined as the ratio of the weight of the composite hydrogels after centrifugation to the customary composite hydrogel.

#### 3.6.3. Thermogravimetric Analysis (TGA)

The thermal properties of the composite hydrogel were measured using a Q50 thermogravimetric analyzer (TA Instrument, New Castle, DE, USA). The samples were weighted and heated from 30 °C to 600 °C with a heating rate of 10 °C/min. The thermal parameters of the composite hydrogel were analyzed.

### 3.7. Statistical Analysis

Statistical analysis was performed using one-way analysis of variance on IBM SPSS statistics (IBM SPSS Inc., Chicago, IL, USA) and presented as mean ± standard deviation. Each sample was tested three times. Significant differences were evaluated by Duncan’s analysis and the level of significance was set at *p* ≤ 0.05.

## 4. Conclusions

In this study, we first investigated the influence of KGM mixed with oat β-glucan on its hydrocolloidal properties. Compared with single hydrogel, KGM/oat β-glucan composite hydrogel synergistically interacted with heating and alkali treatment and was imparted powerful rheological properties and physicochemical properties. The gelation temperatures and viscoelastic modulus of the composite hydrogel were higher than the KGM gel, owing to the enhanced intermolecular force between KGM and oat β-glucan. The gel network of the composite hydrogel was denser after the addition of oat β-glucan, which significantly enhanced the WHC of the composite hydrogel. The composite hydrogel had good thermal stability compared with pure KGM gel, which suggested the composite hydrogel owned the ability to withstand a high temperature environment and maintain its structural integrity. In summary, the above results strongly proved that the addition of oat β-glucan could improve the deficiency of pure KGM gel, and also broaden the application of konjac glucomannan and oat β-glucan in gel food trains of thought.

## Figures and Tables

**Table 1:** The TGA parameters of the KGM gel and the composite hydrogel.

Samples	First-Step Weight Loss (%)	Second-Step Weight Loss (%)	Third-Step Weight Loss (%)	Tmax (°C)
KGM gel	22.51	20.94	8.09	238.75
KOBG-1	22.51	27.74	9.55	239.13
KOBG-2	22.51	31.50	12.39	240.37
KOBG-3	22.51	33.76	12.80	244.73
KOBG-4	22.51	33.78	14.35	259.49
KOBG-5	22.51	37.43	16.75	261.86

Tmax presents the temperatures of the maximum weight loss.

## Data Availability

Not applicable.
